# Modulation of Neuron and Astrocyte Dopamine Receptors via Receptor–Receptor Interactions

**DOI:** 10.3390/ph16101427

**Published:** 2023-10-08

**Authors:** Diego Guidolin, Cinzia Tortorella, Manuela Marcoli, Chiara Cervetto, Raffaele De Caro, Guido Maura, Luigi F. Agnati

**Affiliations:** 1 Department of Neuroscience, University of Padova, 35122 Padova, Italy; cinzia.tortorella@unipd.it (C.T.); rdecaro@unipd.it (R.D.C.); 2 Department of Pharmacy, University of Genova, 16126 Genova, Italy; manuela.marcoli@unige.it (M.M.); cervetto@difar.unige.it (C.C.); guido.maura@gmail.com (G.M.); 3 Department of Biomedical, Metabolic Sciences and Neuroscience, University of Modena and Reggio Emilia, 41121 Modena, Italy; luigi.agnati@gmail.com

**Keywords:** receptor–receptor interactions, receptor complexes, GPCR, dopaminergic pathways, Parkinson’s disease, Schizophrenia, drug addiction

## Abstract

Dopamine neurotransmission plays critical roles in regulating complex cognitive and behavioral processes including reward, motivation, reinforcement learning, and movement. Dopamine receptors are classified into five subtypes, widely distributed across the brain, including regions responsible for motor functions and specific areas related to cognitive and emotional functions. Dopamine also acts on astrocytes, which express dopamine receptors as well. The discovery of direct receptor–receptor interactions, leading to the formation of multimeric receptor complexes at the cell membrane and providing the cell decoding apparatus with flexible dynamics in terms of recognition and signal transduction, has expanded the knowledge of the G-protein-coupled receptor-mediated signaling processes. The purpose of this review article is to provide an overview of currently identified receptor complexes containing dopamine receptors and of their modulatory action on dopamine-mediated signaling between neurons and between neurons and astrocytes. Pharmacological possibilities offered by targeting receptor complexes in terms of addressing neuropsychiatric disorders associated with altered dopamine signaling will also be briefly discussed.

## 1. Introduction

Dopamine (DA) is a catecholamine, that is, an ethylamine with an attached catechol group (a phenyl group with two hydroxyl groups in meta- and para positions). DA-producing neurons were first identified and mapped in animals by Dahlström and Fuxe in 1964 [1,2], indicating the existence of neuronal circuits using DA as a neurotransmitter. In the years that followed, the characterization of the circuits which utilize DA, their organization, molecular signature, and cellular and functional features represented one of the most fertile fields of research in neuroscience (see [3]). Over the past decades, technological advances have also helped to expand the knowledge about the anatomical organization of the DA systems in the human brain. Dopamine neuronal populations have, indeed, been identified and characterized in the human brain from the level of gene transcription to the level of the distribution of related proteins by using post-mortem immunohistochemistry and in vitro autoradiography methods, as well as through in vivo neuroimaging techniques such as positron emission tomography and single photon emission tomography (see [4]). As summarized in Table 1, four major dopamine pathways and two additional ones can be described in the human brain [5,6]. They are involved in the regulation of both physiological and behavioral processes, including movement, endocrine control, cognition, reward, and motivation.

The release of the catecholamine from nerve endings upon axonal stimulation certainly represents the main process of dopamine-mediated interneural communication. Released DA acts on postsynaptic and presynaptic receptors at the synapse and is mostly taken up back into nerve endings by the dopamine transporter protein, which belongs to the solute carrier transporter family [6]. In some regions of the central nervous system, however, dopamine signaling also occurs through processes of “volume transmission”, based on the diffusion of the molecule in the extra-cellular space to reach more distant targets (see [7,8,9]). Examples include globus pallidus [10], substantia nigra [11], ventral tegmental area [12], ventral subiculum [13], pedunculopontine nucleus [14], and retina [15]. In this respect, of significant interest is evidence indicating that DA, interacting with DA receptors expressed by astrocytes [16,17,18], may also act on these cells, leading to a modulation of neuron–astrocyte crosstalk (see [19]).

Dopamine receptors belong to the superfamily of G-protein-coupled receptors (GPCRs). A first indication of their existence was reported in 1972 [20]; they were identified in 1975 [21,22], and five different subtypes have been described so far. In view of the strong implication of DA signaling in a variety of neurological, psychiatric, and drug addiction disorders, with a relevant impact not only on afflicted individuals, but also on society, DA receptors have been the focus of intense research efforts and a variety of drugs have been designed to treat these illnesses by targeting DA receptors directly or indirectly (see [6]).

In recent decades, experimental evidence demonstrating that structural receptor–receptor interactions (RRIs) may occur between receptor proteins has been of interest [23,24,25,26,27,28,29,30,31,32,33]. The term RRI indicates a type of interaction needing a direct physical contact between the partner proteins, with the formation of oligomeric complexes at the cell membrane (see [34] for a recent review). Available studies indicated the formation of receptor complexes as a quite common process in the different receptor families, where the ion channel receptors are at one end of the spectrum (being assembled by multimerization) and GPCRs at the other. Thus, as pointed out by Changeux and Christopoulos in a detailed review [35], RRIs emerge as an efficient mechanism for modulating the functional properties of receptor proteins, including GPCRs that are able to signal as monomers. This mechanism, indeed, allows a sophisticated regulation of the intercellular communication already at the membrane level [9] and opens the possibility of new pharmacological strategies to modulate receptor signaling. In this context, several groups (including our group), have focused their attention on the detection of receptor complexes containing DA receptors in nervous tissues and on the role they can play in DA-mediated signaling in neurons and astrocytes. In the present review, published data concerning this modulatory process will be presented and discussed. Since the subject is quite broad, review articles focused on specific aspects of the topic will also be suggested for further information.

## 2. Dopamine Receptors

Five different subtypes of DA receptors (D_1_, D_2_, D_3_, D_4_, and D_5_) have been identified in brain tissue (see [6] for a recent review), and based on their structure and pharmacological properties, they can be classified into two major groups [36]: D_1_-like receptors (including D_1_ and D_5_) and D_2_-like receptors (comprising D_2_, D_3_, and D_4_). Binding studies have demonstrated some differences between the two groups in terms of affinity to DA, with D_2_-like receptors exhibiting a 10- to 100-fold greater affinity to DA than D_1_-like receptors [37,38,39]. D_1_- and D_2_-like receptors also differ in their genetic structure. D_2_-like receptor genes, indeed, have introns in their coding regions, while D_1_-like receptor genes do not exhibit this feature [40]. This genetic organization, therefore, enables the generation of D_2_-like receptor splice variants, and alternative splicing is particularly important for the D_2_ receptor, leading to the generation of two distinct receptor isoforms: D_2_-short and D_2_-long [41,42], differing because of the insertion of 29 amino acids in the D_2_-long intracellular domain, which may play a role in determining second messenger specificity [36,42].

Concerning signal transduction, it is commonly accepted that the receptors of the D_1_-like group mainly mediate the stimulation of the second messenger adenylyl cyclase (cAMP) by coupling to the G_s_ protein, whereas receptors of the D_2_-like group mainly exert inhibitory effects on this enzyme by coupling to G_i/0_ protein [6,43]. In addition to the just mentioned main pathway, D_1_-like receptors may also couple to the G_q_ protein [44,45,46] and modulate phospholipase C [44,46,47], leading to an increase in intracellular calcium levels and activation of protein kinase C. In this respect, the regulation of intracellular calcium levels is a well-documented action of dopamine on astrocytes [48]. DA receptors are expressed by astrocytes [49], and D_2_ receptor activation was reported to decrease intracellular Ca^2+^ levels in hippocampal [50] and ventral midbrain astrocytes [51], while D_1_ receptor activation elevated astrocytic Ca^2+^ levels in the hippocampus [50], nucleus accumbens [52], and cerebellum [53].

DA signaling cascade, however, may also be modulated by the significant network of molecular interactions that DA receptors can establish in their environment [6,43], which interfere with the GPCRs activity. A first example [54,55] is provided by G-protein-coupled receptor kinases (GRKs). GRKs phosphorylate receptors in response to persistent stimulation [56]. Consequently, the receptor becomes a target for a scaffolding protein, named arrestin, blocking further activation of the GPCR [57] and allowing the GPCR–arrestin complex to engage a variety of G-protein-independent signaling pathways [58]. A second example [59,60] is represented by the regulators of G protein signaling (RGS). RGS are a family of more than 35 intracellular proteins (see [6]) that induce inhibitory effects on GPCRs. Concerning DA receptors, they mainly regulate the D_2_-like class [61] and are important in order to stop signaling in the slow synaptic transmission elicited by D_2_ receptors [38,62].

In this context, of particular interest is the possibility of direct RRI involving DA receptors with the formation of receptor complexes at the cell membrane [63,64]. In receptor complexes, indeed, the chain of events linking the recognition of a ligand by the single protomers to the signal transduction also depends on the neighboring receptors. This specific mechanism modulating DA signaling will be the focus of the next sections.

## 3. Structural Receptor–Receptor Interactions

Functional interactions between receptors, by mechanisms of transactivation or by sharing signaling pathways, are well-known processes that do not need a physical contact between the involved proteins [65]. In the 1980s, however, Agnati, Fuxe, and collaborators [23,66], through in vitro and in vivo experiments, provided indirect evidence that GPCR monomers can establish structural interactions (see [63] for historical details). These findings led to the hypothesis that neuron activity could be modulated by receptor complexes present at the cell membrane and formed by different types of GPCRs [64], a mechanism allowing (already at the membrane level) some integration of synaptic (wiring transmission) and extra-synaptic (volume transmission) signals [64]. The term RRI was subsequently proposed to emphasize the concept of an interaction between receptors requiring a direct physical contact between the molecules and leading to the formation of dimers or high-order molecular complexes at the cell membrane [67]. In the years that followed, several groups [23,24,25,26,27,28,29,30,31,32,33] provided direct evidence of the existence of this structural organization, and the amount of data supporting the existence of GPCR complexes further increased with the advent of biophysical techniques capable of detecting the spatial proximity of protein molecules [68,69,70,71]. The obtained results demonstrated that GPCRs can signal not only as monomers, but also as part of receptor complexes [72] and indicated that receptor complexes represent a quite common molecular organization in the different families of receptors [34].

The basic molecular mechanism underlying the formation and the dynamics of these receptor assemblies are allosteric interactions (see [73]). Allostery (see [35,74,75,76] for extensive reviews) is a mode of communication between distant sites in a protein, in which the energy associated with dynamic or conformational changes at one site can be transferred (along specific pathways within the protein structure) to other sites, that, in turn, will change their conformational or dynamic features. Thus, when a quaternary structure is established via direct RRIs between protomers, energy perturbations at some site of one protomer can propagate into the nearby protomers and change their conformational and functional properties, leading to a cooperative behavior of the whole complex [34,77]. In current research on receptor oligomerization, therefore, the identification of the residues forming the interface between protomers is of significant interest. They, indeed, influence the overall architecture that the receptor complex can assume. In this respect, to predict the interfaces available for RRIs, several bioinformatics methods have been developed (see [78,79,80] for reviews on this topic). As a matter of fact, the number of ways GPCRs interact in the membrane to form complexes is probably limited. The vast majority of experimentally identified receptor complexes, indeed, are dimers. And some interfaces have been observed to be more exploited than others for RRIs [81]. Nevertheless, oligomeric heteroreceptors have been detected (see [81,82,83,84]).

**Figure 1:**
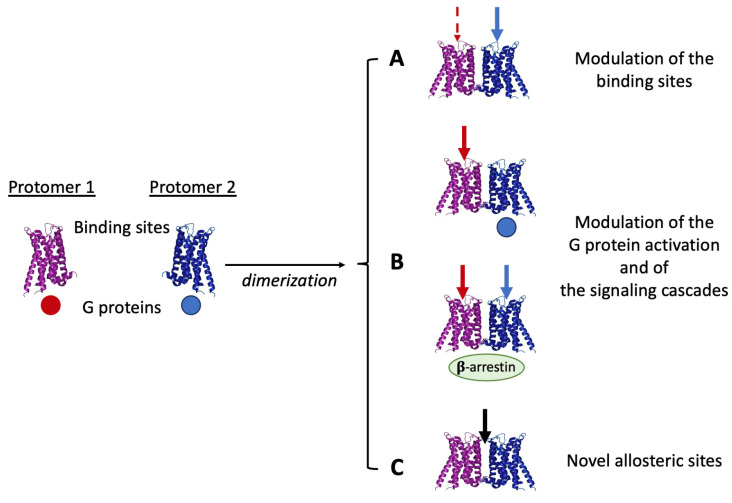
As a result of allosteric RRIs, receptor complexes appear to be endowed with pharmacological features that cannot be fully derived from the characteristics of the single participating protomers (see text).

The signaling outcomes from a receptor complex, therefore, depend on several factors (including the composition and the topological organization of the complex and the effects of ligands on its stability and trafficking), which may strongly influence the cascade of events linking the recognition of a ligand by single protomers to the signal transduction (see [43,80,85]). Some of the possible modulations that allosteric RRIs may induce on signaling when a receptor complex forms are summarized in Figure 1. They include changes in ligand recognition, G-protein activation, receptor desensitization [86], and switching to β-arrestin signaling [87]. In this context, a relevant aspect of receptor complex formation is also the possible appearance in the formed quaternary structure of novel specific allosteric sites allowing the binding of some modulator. Thus, ligands specific to the receptor complex as such may also exist (see [88]).

A final aspect deserving consideration (see [34] for a discussion) concerns the cell environment in which receptor complexes are located. In fact, the network of molecular interactions they can establish at the cell membrane with other biochemical components (the so-called “horizontal molecular networks” [89]) may influence their signaling. In this context, a specific aspect of interest is the lipid environment, since it was shown to influence receptor function [90]. In particular, changes in the membrane composition altering receptor signaling were associated with several health disorders during aging [90].

## 4. Receptor Complexes Involving Dopamine Receptors

DA receptors belong to class A GPCRs [91], well known for being able to signal as monomers [92]. In addition, however, the overall available evidence (obtained through multiple approaches with consistent results) strongly supports the presence of class A GPCR complexes in native systems [72]. In this respect, studies concerning the kinetics of complex formation and its dependence on the involved interaction energy [93] are of substantial interest. The observed half-lives of dimers indicate that they are often transient (lasting few hours) and may undergo recombination (“kiss-and-run” encounters [80]). These processes may lead to a dynamic equilibrium between monomers and receptor complexes for class A GPCRs, as suggested by studies on the corticotropin-releasing factor receptor type 1 in the endoplasmic reticulum [94], indicating that the ratio of monomers/receptor complexes was maintained at an almost constant level in the plasma membrane, even in spite of agonist activation of the receptors. Receptor complexes including DA receptors (see also [95]) are shown in Table 2.

### 4.1. Receptor Complexes Involving D_2_-like Dopamine Receptors

A first aspect emerging from the available data is that D_2_ appears to be a hub receptor which interacts with many other GPCRs.

Probably, the most studied interaction is between dopamine D_2_ and adenosine A_2A_ receptors, leading to the formation of A_2A_-D_2_ heterodimers (see [95,120] for reviews). By using pull-down and mass spectrometry techniques, it has been demonstrated [121,122] that the heteromerization between A_2A_ and D_2_ receptors significantly depends on charged residues located at the intracellular part of the transmembrane helix 5 (TM5) of the D_2_ receptor. The role of TM helix interactions within the A_2A_-D_2_ heteroreceptor complex interface has also been explored by using synthetic TM α-helix peptides of the D_2_ receptor [123], and the results allowed for the identification of a TM4/5 interface between the two monomers. The A_2A_-D_2_ heterodimer is also representative of many aspects concerning the signaling outcome from a receptor complex. Experimental evidence has shown that the receptor complex formation modifies the signaling from the single protomers. In particular, early in vitro experiments on membrane preparations showed a reduction in the affinity of the high-affinity D_2_-agonist-binding site after incubation with the A_2A_ agonist CGS21680 [124,125], demonstrating that antagonistic interactions occur in the A_2A_-D_2_ heterodimer. By using receptor autoradiography, this finding was subsequently confirmed by studies on brain tissue from rats and humans [126]. They showed a strong reduction in D_2_ receptor affinity for dopamine in the nucleus accumbens core and shell after the A_2A_ receptor agonist treatment. By using functional, biochemical, and biophysical techniques (such as co-immunoprecipitation and proximity ligation assay), antagonistic interactions between A_2A_ and D_2_ receptors were also recently demonstrated in astrocytes [96,127]. In this context, observations indicating that agonist activation of the A_2A_ protomer in the A_2A_-D_2_ heteroreceptor complex inhibits D_2_ G_i/o_-mediated signaling but increases the D_2_ β-arrestin_2_-mediated signaling are of interest. This marks a difference compared with the action of D_2_ receptor antagonists, which block all the D_2_ signaling pathways. Thus, through the allosteric receptor–receptor interaction, an A_2A_ agonist becomes a biased inhibitory modulator of the G_i/o_-mediated D_2_ signaling [128]. The possible formation, as a consequence of the formation of a receptor complex, of new allosteric sites allowing the binding of some ligand is a further modulatory mechanism that the A_2A_-D_2_ heteromer illustrates. Homocysteine can, indeed, bind to the heterodimer without interfering with the RRI between A_2A_ and D_2_ and acts as an allosteric antagonist of the D_2_ receptor [129]. Thus, the inhibitory effect of A_2A_ agonists is amplified by homocysteine. These modulatory actions were demonstrated in striatal neurons [129], as well as in astrocytes [130], where homocysteine reduces the D_2_-mediated inhibition of glutamate release. An intriguing process involving A_2A_ and D_2_ receptors was highlighted by studies on cell lines [19] that demonstrated intercellular transfer of these GPCRs by exosomes, resulting in the incorporation of functional receptors into acceptor cells. As shown by photo-bleaching fluorescence resonance energy transfer, the transferred receptors may also undergo A_2A_-D_2_ receptor heteromerization in the target cell. Thus, the release of extracellular micro vesicles (the so-called “roamer type” of volume transmission [19]) may represent a significant mechanism for the modulation of neuron-neuron and astrocyte–neuron intercellular signaling.

Evidence has been provided indicating that the adenosine A_2A_ receptor can establish antagonistic RRIs with the other D_2_-like receptors as well, namely, D_3_ [97] and D_4_ [98], leading to a reduction in the affinity of their binding site for DA. Antagonistic RRIs also characterize other receptor complexes involving the D_2_ receptor, as, for instance, the heterodimers it can form with the glutamate NMDA [99] and mGluR_5_ [84] receptors, the neurotensin NTS_1_ [100] receptor, and the cannabinoid CB_1_ [102,131] receptor. Higher-order heteroreceptor complexes, involving both A_2A_ and D_2_, have also been identified. Examples include the heterotrimers formed by A_2A_ and D_2_ receptors with the metabotropic glutamate receptor 5 (A_2A_-D_2_-mGluR_5_ [84]), the sigma_1_ receptor (A_2A_-D_2_-sigma_1_ [113]), and the cannabinoid CB_1_ receptor (A_2_A-D_2_-CB_1_ [82]). In these receptor complexes, the pattern of allosteric interactions on the D_2_ protomer also inhibits the recognition and signaling of the DA receptor.

Synergistic RRIs involving the D_2_ receptor, however, were also identified. A first example is provided by the receptor complex between the D_2_ receptor and the serotonin 5-HT_2A_ receptor [104], where the activation of the 5-HT_2A_ protomer by 5-HT_2A_ agonists produced an enhancement of D_2_ signaling. In astrocytes, receptor complexes between the dopamine D_2_ receptor and the serotonin 5-HT_1A_ receptor have been observed [103]. However, the functional consequences of the signaling pathways mediated by D_2_-5-HT_1_ heteromers in these cells are still not known in detail [132]. A further example is represented by the D_2_-OTR heterodimer, involving D_2_ and the oxytocin receptor. In neurons [105], oxytocin, via the allosteric RRI established in the heterocomplex, markedly increased D_2_ receptor recognition (increased affinity of the high-affinity state) and increased the coupling of G_i/o_ to the receptor. The D_2_-OTR heterodimer was recently identified in astrocytes as well [106], and the activation of OTR was shown to have a facilitatory effect on the response of D_2_ receptors, causing them to be activated by subthreshold D_2_ agonist concentrations and leading to an inhibition of glutamate release by the cells.

Synergistic RRIs are also in operation in the heterodimer involving the dopamine D_4_ receptor and μ-opioid receptor (MOR) [110], since D_4_ activation causes a substantial increase in the affinity of the MOR agonist binding sites. Evidence was also obtained that the D_4_ and β_2_-adrenergic receptor may form a D_4_-β_2_ receptor complex that integrates G_s_- and G_i_-mediated regulation of adenylyl cyclase [109]. In this context, of particular interest are also studies (see [108]) focused on the dopamine D_4_ receptor polymorphic variants D_4.4_ (four repeats in exon 3) and D_4.7_ (seven repeats in exon 3), both able to heterodimerize with the norepinephrine α_2A_ receptor. However, only heteromerization with D_4.7_, but not with D_4.4_, increases the potency of norepinephrine in terms of activating the α_2A_ receptor, indicating the possible polymorphic variants of a D_2_-like receptor as a factor conferring significantly different pharmacological properties onto the receptor complexes it may form.

### 4.2. Receptor Complexes Involving D_1_-like Dopamine Receptors

The potentiation of immediate early gene expression and of arachidonic acid release have been described as functional interactions between activated dopamine D_1_ and D_2_ receptors (see [45]). However, it was also demonstrated that stably co-expressed D_1_ and D_2_ receptors may form heteromeric units [114]. It is of substantial interest that the two receptors, when coactivated in the same cell, produce a phospholipase C-mediated calcium signal that is not seen when the receptors are activated alone. The pharmacological analysis of this receptor complex indicated a specific coupling to the G_q/11_ pathway to produce such a response. Activation of G_q/11_, however, could not be elicited through activation of either receptor when activated alone. Thus, the recruitment of G proteins other than those expected for the monomers has been observed after D_1_-D_2_ dimerization, a further mechanism of signal transduction modulation associated with receptor complex formation.

Antagonistic interactions between D_1_ and the adenosine A_1_ receptor, associated with the formation of A_1_-D_1_ heterodimers [116,117], were also characterized. A_1_ agonists, indeed, were found to reduce the number of D_1_ agonist binding sites in the high-affinity state, and with receptor autoradiography, A_1_ agonists were found to antagonistically modulate D_1_ binding sites, causing a reduction in their affinity (see [133] for details).

Receptor complexes between dopamine D_1_ and D_3_ have been demonstrated using several techniques, giving evidence for synergistic intramembrane D_1_-D_3_ interactions at the level of D_1_ recognition, since D_3_ activation was able to increase the affinity of the D_1_ agonist binding sites [115]. Synergistic RRIs also exist in the D_1_-NMDA heterodimer [118], by which NMDA receptor activation can recruit D_1_ receptors to the plasma membrane, thereby leading to an increase in D_1_ signaling and cAMP accumulation.

Recent interesting findings on prefrontal cortex astrocytes indicated a significant functional interaction between α_1_-adrenergic and DA receptors, driving downstream Ca^2+^ signaling [112]. Also, in light of the abovementioned data showing that DA receptors may form receptor complexes with adrenergic receptors [109,134], and of neuroanatomical data showing that D_1_ and α_1_-adrenergic receptors colocalize on prefrontal cortex dendrites and may undergo co-trafficking [119], the hypothesis has been put forward that in cortical astrocytes as well, heterodimers involving DA receptors and adrenergic receptors could be present [112]. A direct experimental demonstration, however, is still lacking.

GABA_A_ and dopamine D_5_ heteromerization, demonstrated by Liu and collaborators [135], was the first identification of a receptor complex involving a GPCR and an ion-channel receptor. The results indicated that co-activation of the monomers was required for the formation of the complex, which allowed for a bidirectional crosstalk, leading to a reduction in GABA_A_ signaling and a reduced coupling between D_5_ and G_s_ proteins.

### 4.3. Possible Differences in Receptor Complex Dynamics in Neurons and Astrocytes

As briefly illustrated before, a number of receptor complexes (such as, for instance, the A_2A_-D_2_ heterodimer) are expressed both in neurons and astrocytes. In this respect, it is reasonable to assume that the conformation of a receptor complex in the two cases may exhibit some difference because of differences in the membrane microenvironment. Differences in the energy landscape, indeed, modulate the pattern of allosteric interaction between monomers and may lead to changes in the signaling features of the complex that they can form [80].

Differences in membrane potential between the two cell types, for instance, have been documented [136]. Unlike neurons, astrocytes do not generate action potentials, but they are electrically dynamic cells. Indeed, in contrast to most non-excitable cells that have relatively depolarized membrane potentials, astrocytes have a hyperpolarized membrane (at a level that typically rests significantly below that of neurons) and a low membrane resistance. For the present discussion, membrane composition is another factor deserving consideration. This aspect was the focus of an extensive lipidome analysis by Fitzner and collaborators [137], showing that each cell type was characterized by a unique lipid composition: neurons, for instance, exhibited quite high levels of cholesterol, while astrocytes were enriched in phosphatidylinositol.

**Figure 2:**
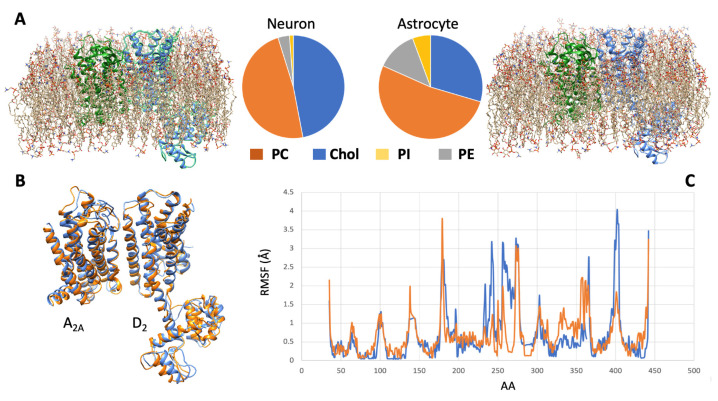
Molecular dynamics simulation of the A_2A_-D_2_ receptor complex in different cell membranes. (**A**) By using the CHARMM-GUI membrane builder web server (http://www.charmm-gui.org/?doc=input [138], accessed on 5 July 2023), four phospholipids, namely, phosphatidylcholine (PC), cholesterol (Chol), phosphatidylinositol (PI), and phosphatidylethanolamine (PE), were used to model two different membrane bilayers around the molecular model [120,123] of the heterodimer. The first (left panel) approximated the neuronal membrane composition, the second one (right panel) the astrocytic one (see [137]). A molecular dynamics procedure, based on the CABSflex method [139] and available as a web server (https://biocomp.chem.uw.edu.pl/CABSflex2, accessed on 6 July 2023), was then used to evaluate the conformations that the receptor complex may acquire in the two environments. (**B**) Configurations of minimal energy of the A_2A_-D_2_ heterodimer in neuronal (orange) and astrocytic (blue) membrane. (**C**) Root mean square fluctuations (RMSF) diagrams, per amino acid position, of the D_2_ monomer chain when in neuronal (orange) and astrocytic (blue) membrane. The estimated differences in configuration and dynamical behavior of the heterodimer suggest that different membrane environments could represent a factor modulating the pharmacological properties of the receptor complex.

All these features of the membrane microenvironment, therefore, have the potential to modulate the pharmacological properties of a given receptor complex. To illustrate this concept, the results of a simulation based on molecular modeling methods and focused on the A_2A_-D_2_ heterodimer in two different membrane environments (neuron-like and astrocyte-like) are shown in Figure 2.

## 5. Complexes Involving Dopamine Receptors in the Main Dopaminergic Pathways: Impact on Neuropharmacology

The intermingling of findings from functional neuroanatomy (linking dopaminergic pathways to specific functions and diseases) with evidence emerging from chemical neuroanatomy (describing the distribution of receptor complexes involving DA receptors in brain cells and regions) may help to better appreciate the function that receptor complexes containing DA receptors can fulfill, and may contribute to the development of new pharmacological approaches with a potentially major impact on molecular medicine. In this respect, presently available information is limited to ascending dopaminergic pathways (nigro-striatal, mesolimbic, and mesocortical) and neuron–astrocyte crosstalk, being descending pathways (mentioned in Table 1) almost uninvestigated in terms of receptor complexes containing DA receptors. Thus, in the sections that follow, only the abovementioned signaling pathways will be considered (see also [108,133,140,141,142] for reviews). These are, however, of significant interest, being associated with an impact on neuropsychiatric diseases. Reported findings are summarized in Table 3.

### 5.1. Nigro-Striatal Dopamine Pathway

The nigro-striatal pathway starts from dopamine-containing cells in the substantia nigra pars compacta (SNc) of the midbrain to establish multiple synaptic contacts with medium spiny neurons (MSNs) of the ipsilateral dorsal striatum [143]. MSNs also receive cortico-striatal glutamatergic afferents and are GABAergic projection neurons classified into three populations [3]. Island (patch) MSNs are localized in the so-called striatosomes [144] and send a feedback signal to neurons of the SNc, striato-nigral/entopenducular MSNs project to the substantia nigra pars reticulata (SNr) and the entopeduncular nucleus (EPN) (nuclei from which the so-called direct pathway of motor control starts), and striato-pallidal MSNs project to the external globus pallidus (GPe) (nucleus from which the indirect pathway of motor control starts), which in turn modulates the subthalamic nucleus (STh). The direct pathway triggers a disinhibition of the target regions, whereas the indirect pathway triggers their inhibition, leading to activation and suppression of motor behavior, respectively. In terms of the dopaminergic modulation of these pathways, the direct pathway is dominated by D_1_ receptors, expressed at a high level by striato-nigral/entopeduncular MSNs, while the indirect pathway is mainly regulated by D_2_ receptors, well expressed by striato-pallidal MSNs [3].

As an endogenous neuroprotectant agent, adenosine is extensively distributed in the central nervous system, where it acts trough specific receptors [145], and in the dorsal striatum, A_1_ and A_2A_ adenosine receptors are widely expressed in both MSNs [133] and glutamatergic terminals [134]. It is not surprising, therefore, that receptor complexes involving adenosine and dopamine receptors were identified in the dorsal striatum. In striato-nigral/entopeduncular MSNs, for instance, the presence of the A_1_-D_1_ heterodimer has been reported [116,146], while receptor complexes involving the adenosine A_2A_ and the D_2_ receptors (namely, the A_2A_-D_2_ heterodimer and the heterotrimers A_2A_-D_2_-mGluR_5_ and A_2A_-D_2_-CB_1_) were found in striato-pallidal MSNs and their glutamate inputs [82,84,95]. STh is also innervated by collaterals of the nigro-striatal bundle [143], and co-localization of A_2A_ and D_2_ receptors has been recently documented in this nucleus [147], opening the possibility of the presence (yet to be substantiated) of A_2A_-D_2_ heterodimers within the dorsal and medial aspects of the structure.

**Figure 3:**
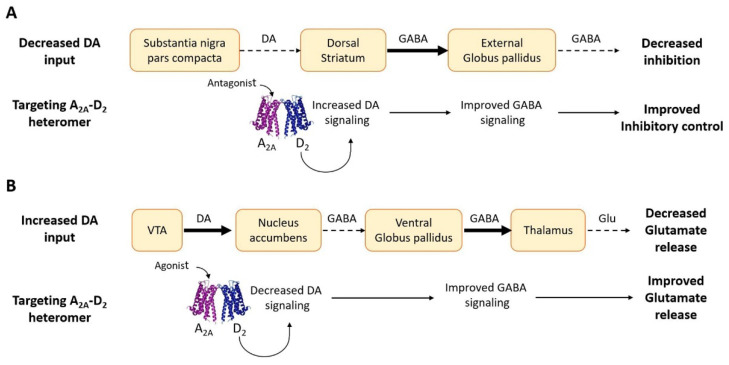
Schematic representation of pharmacological strategies to address imbalance of DA signaling by targeting the A_2A_-D_2_ receptor complex [120]. (**A**) Decreased DA signaling in the nigro-striatal pathway (as in Parkinson’s disease) leads to a reduced D_2_ activity and to a decreased inhibitory output from the external globus pallidus to the downstream structures, resulting in unbalanced motor control. Targeting A_2A_-D_2_ heteromers in the striatum with antagonists of the A_2A_ receptors may improve D_2_-mediated dopaminergic signaling and motor control. (**B**) Overactivity of the mesolimbic dopamine neurons increases the D_2_-mediated dopamine transmission to the ventral striatum, leading to a reduced glutamate drive from the mediodorsal thalamic nucleus. A_2A_ agonists targeting the antagonistic interactions between A_2A_ and D_2_ receptors in the complex may improve this condition. Dashed arrows and thick arrows indicate decreased and increased signaling, respectively.

Parkinson’s disease (PD) is a common disease, associated with neurodegeneration of the nigro-striatal pathway, leading to imbalance or loss of dopaminergic signaling to the dorsal striatum with the emergence of altered motor features, such as bradykinesias, tremor, and rigidity. The introduction of L-DOPA [148] revolutionized the management of this disease, leading to an effective symptomatic treatment. However, it soon became apparent that the drug offered only symptomatic relief and did not affect the underlying pathology. Moreover, chronic use of the drug was associated with a range of adverse effects, such as dyskinesias, toxicity, or loss of efficacy [149]. Current therapeutic protocols, therefore, seek to delay long-term complications of treatment for as long as possible. In this context, the antagonistic allosteric RRIs described earlier, which characterize the receptor complexes involving adenosine and dopamine receptors, led to the hypothesis (schematically illustrated in Figure 3A) that by targeting these heteromers with antagonists of the adenosine receptors, antiparkinsonian effects could be obtained (see [150] for a specific review on this topic). This research effort mainly focused on A_2A_-D_2_ receptor complexes. Animal models of PD gave support to the hypothesis and clinical evidence was also obtained (see [120] for references). In this respect, it is of interest to mention the very recent approval in the United States of an A_2A_ antagonist (istradefylline) as an adjunctive treatment to L-DOPA [151] in PD. Following the same logic, D_1_ signaling in the A_1_-D_1_ heterodimer could be modulated by targeting the adenosine A_1_ receptor to obtain antiparkinsonian effects [133].

Other receptor complexes in the dorsal striatum, however, deserve a mention as possible pharmacological targets in PD. CB_1_ antagonists targeting the CB_1_-D_2_ heterodimer, for instance, may represent possible antiparkinsonian drugs, since the antagonistic RRIs, characterizing this receptor complex, can enhance D_2_ signaling [152]. Behavioral correlates to the antagonistic receptor interactions in CB_1_-D_2_ heterodimers have also been obtained using the CB_1_ receptor agonist HU-210, which has been found to reduce L-DOPA-induced rotations in 6-hydroxydopamine-lesioned rats [153]. In cortico-striatal glutamate terminals, the D_2_-NMDA receptor complex (with antagonistic RRI) is constitutively present [99] and inspired the possibility that a dual approach in PD with low doses of selective D_2_ agonists and NMDA antagonists could lead to antiparkinsonian actions with reduced development of dyskinesias [133].

### 5.2. Mesolimbic Dopamine Pathway

The mesolimbic pathway connects the ventral tegmental area (VTA), a dopaminergic nucleus of the midbrain, with the ventral striatum (occupying about 20% of the striatum), including the nucleus accumbens (NAc) and the olfactory tubercle, which are striatal regions receiving their major telencephalic input from the hippocampal formation and amygdala, and projecting to the ventral pallidum (VP) and SNr. From there, information is transferred to the anterior cingulate cortex and the orbitofrontal cortex [154]. Concerning the NAc, two main subterritories have been identified, namely, the shell and the core, the shell region being more closely associated with the limbic system than the other regions of the ventral striatum [3].

Ventral striatum neurons are MSNs, similar to those of the dorsal striatum, and their dopaminergic input are mainly regulated by D_2_ receptors [155]. A_2A_-D_2_ heteroreceptor complexes with antagonistic RRIs were demonstrated in the ventral striatum [125], as were high-order receptor complexes including adenosine A_2A_ and dopamine D_2_ receptors, such as, for instance, the A_2A_-D_2_-mGluR_5_ and A_2A_-D_2_-sigma_1_ heterotrimers [156]. Of interest is also the presence in ventral MSNs of cortico-accumbens terminals of receptor complexes involving dopamine D_2_, glutamate NMDA [99], neurotensin NTS_1_ [100], serotonin 5-HT_2A_ [104], and oxytocin [105] receptors.

The mesolimbic pathway is a key element of the so-called reward circuit (see [154]), because the release of dopamine through this pathway regulates motivation and desire for rewarding stimuli (i.e., incentive salience), facilitates reinforcement- and reward-related motor function learning, and may also play a role in the subjective perception of pleasure. Thus, the dysregulation of the mesolimbic pathway and its downstream neurons plays a significant role in the development of significant neuropsychiatric diseases, including addiction and schizophrenia (see [156,157] for specific reviews).

A study [158], for instance, showed that chronic cocaine self-administration increased behavioral responses mediated by D_2_ receptors, indicating the relevance of D_2_ for cocaine use disorder. Furthermore, chronic cocaine self-administration persistently evoked more than 100% elevations of D_2_ binding sites of the high-affinity type [159], and D_2_ activation produced a strong relapse of cocaine seeking in animals [160]. In this respect, studies focused on the antagonistic RRIs in the A_2A_-D_2_ receptor complex as a possible pharmacological target indicated that A_2A_ agonists exhibited an inhibitory effect on cocaine reward [160], and A_2A_ activation, leading to D_2_-like receptor blockade, counteracted cocaine relapse. It is also of interest that cocaine induces a selective increase in sigma_1_ receptors in the ventral striatum [161]. Thus, the A_2A_-D_2_ antagonistic interaction may become more present thanks to a higher presence of A_2A_-D_2_-sigma_1_ receptor complexes. In this context, also results suggesting the existence of D_4_-MOR heterodimers [110] in the striatosomes and SNr, in which D_4_-MOR interactions are in operation, are also of interest. They may play a critical role in at least the early stages of the expression of the morphine effects. In view of the limbic-prefrontal–striatosome-nigral circuitry and its function (see [162]), this interaction may participate in reward-based motor learning and play a significant role in habit acquisition in drug addiction [133].

In schizophrenia, salience becomes exaggerated due, inter alia, to an increased D_2_ recognition and signaling in the ventral striatum (mainly nucleus accumbens) [163]. Thus, the classic treatment [164] in schizophrenia is the use of DA receptor antagonists, typically haloperidol and chlorpromazine. Through the blockade of excessive D_2_-mediated DA transmission in the mesolimbic dopaminergic pathway, they allow an improvement of mental symptoms, but induce motor side effects due to the parallel block of the nigrostriatal pathway. Thus, based on the presence in the ventral GABAergic MSNs, in astrocytes, and in glutamatergic terminals of A_2A_-D_2_ containing heteroreceptor complexes with antagonistic A_2A_-D_2_ interactions, the use of A_2A_ receptor agonists (see Figure 3B) as a strategy for the treatment of schizophrenia has been proposed [133] and promising results in animal models have been found [165]. It is worth noting that A_2A_ agonist treatment, especially in combination with low doses of typical and/or atypical D_2_ antagonists, could also represent a possible strategy for reducing the development of extrapyramidal side effects [133]. Facilitatory RRIs in the 5-HT_2A_-D_2_ receptor complex may represent a further target for treatments based on antagonists of the serotonin receptor (see [157]), and a reduction in the inhibitory D_2_ signaling at the cortico-accumbens glutamatergic terminal level could be obtained by targeting NTS_1_-D_2_ receptor complexes with agonists of the neurotensin receptor [101]. The D_2_-OTR heterodimer also deserves interest as a possible target in schizophrenia. Indeed, evidence was obtained that the molecular mechanism mediating the social salience was the formation of D_2_-OTR heteroreceptor complexes in the nucleus accumbens core [105]. In fact, being located to a special component of the ventral GABAergic MSNs involved in regulating a brain circuit reaching into the prefrontal cortex, the result of the activation of the D_2_-OTR heteroreceptor complex may produce social attachment and trust and the negative symptoms of schizophrenia may become markedly reduced [140]. Consistent with this hypothesis are data showing that oxytocin can induce antipsychotic actions [166], which appears to be true after being given to schizophrenic patients intranasally [167].

### 5.3. Mesocortical Dopamine Pathway

The mesocortical pathway connects the VTA to the prefrontal cortex, but dopaminergic axons branch within the cortex to reach multiple cortical areas [3]. By applying a modified Falck–Hillarp technique, Hökfelt and coworkers [168] identified a plexus of dopaminergic fibers in the limbic cortex with an uneven innervation of the entorhinal cortex. DA-containing varicosities preferentially establish synaptic contacts on pyramidal neurons [169].

This pathway is essential to the normal cognitive function of the dorsolateral prefrontal cortex (part of the frontal lobe) and is thought to be involved in cognitive control, motivation, and emotional response [170]. In this respect, it is closely associated with the mesolimbic pathway.

As recently discussed by Ferré and collaborators [108], an interesting aspect of this innervation pattern is the high expression of dopamine D_4_ receptors in the cortex of mammals: most glutamatergic pyramidal neurons and about half of the GABAergic interneurons express D_4_. Considering the G_i_-coupled D_4_ as mostly inhibitory, the D_4_ localized in neurons should be expected to exhibit an inhibitory effect on dopamine, while those localized in GABAergic interneurons should be expected to produce disinhibition. Several studies, however, indicate a more complex picture, associated with evidence indicating that D_4_ receptors can form receptor complexes with adrenergic receptors [171]. As briefly discussed in Section 4.1, these receptor complexes may have significantly different pharmacological properties depending on the polymorphic variant of the D_4_ receptor involved.

In this respect, available evidence associating D_4_ polymorphisms with individual differences in impulse control-related neuropsychiatric disorders is of interest, with the most consistent associations found between the gene encoding D_4.7_ and attention-deficit hyperactivity disorder (ADHD) [172]. On this basis, it has been proposed that receptor complexes involving the D_4_ receptor should be investigated as possible therapeutic targets for ADHD, as well as for restless legs syndrome [108].

### 5.4. Neuron–Astrocyte Crosstalk

Increasing evidence (see [141] for a specific review) indicates that astrocytes are directly involved in the regulation of neuronal excitability and action potential propagation. According to this view, a bidirectional relationship exists between astrocytes and neurons, where neural activity influences astrocytic activation, which in turn modulates the activity of neurons [173].

Astrocytes, indeed, monitor the extracellular environment through specific receptors, including many neurotransmitter receptors (such as those for DA). Single astrocytes integrate this information through the elevation of intracellular Ca^2+^ [141] and can propagate this information over large distances by communicating with each other through calcium waves [174]. Such calcium dynamics are considered a key step leading to the release of gliotransmitters (D-serine, ATP, and glutamate) that regulate ongoing neural activity [175]. As indicated by several experimental studies (see [173] for a review), this intercellular crosstalk significantly influences synaptic plasticity and, consequently, higher CNS functions such as, for instance, learning and memory.

In this context, extensive available data indicate that RRIs may play a significant role. Relevant examples include the heterodimers A_2A_-D_2_ and D_2_-OTR [96,106], formed by the association of the dopamine D_2_ receptor with the adenosine A_2A_ or the oxytocin receptor, respectively. These receptor complexes are present in astrocytes and regulate the release of glutamate from these cells [106,127], a process relevant for the control of glutamatergic transmission in striatum and with potential roles in the dysregulation of glutamatergic transmission in various neuropsychiatric diseases (see [176] for a specific review on this topic).

The results of a study [177], showing that knocking down the striatal astrocytic glutamate transporter GLT-1 induces PD-like changes in rodents, illustrate the importance of the regulation of the striatal extracellular glutamate level by astrocytes in this pathology. Furthermore, dopamine-mediated glutamate release from striatal astrocyte processes can modulate the activation of NMDA and metabotropic glutamate receptors on striatal MSN [178], suggesting the abovementioned receptor complexes as potential targets to counteract striatal glutamatergic transmission disfunctions and circuit derangement in PD [176]. In this respect, an interesting possibility was suggested by findings showing that homocysteine (an allosteric modulator of the A_2A_-D_2_ heterodimer, see Section 4.1) was able to counteract the DA-mediated inhibition of glutamate release by astrocytes [130]. The relevance of this finding from a physio-pathological standpoint can be appreciated when considering that L-DOPA treatment can trigger synthesis of homocysteine in astrocytes and their release into the extracellular space [179].

Evidence indicating astrocyte involvement in schizophrenia has also been collected [180,181], where glial abnormalities were proposed to contribute to glutamatergic and dopaminergic neurotransmission dysfunctions [182]. In a mouse model of astrocytic A_2A_ receptor knockout, for instance, impaired glutamate homeostasis associated with enhanced behavioral sensitization to psychoactive drugs and reduced working memory (two behavioral symptoms of the pathology) was reported [180]. Thus, the astrocytic A_2A_-D_2_ heteromers may represent a possible target for A_2A_ agonist or other drugs (see [183,184]) in order to ameliorate the impaired glutamate homeostasis in schizophrenia.

Regulation of astrocytic RRIs involving the D_2_ receptor can also be of importance for the pathophysiology and treatment of drug addiction. Accumulating evidence, indeed, indicates that drugs of abuse can trigger glutamatergic dysregulation through astroglial mechanisms (see [185]). On this background, D_2_-containing heteromers in astrocytes may provide new perspectives in the search for drug addiction therapies.

## 6. Concluding Remarks

Since the discovery of DA as a neurotransmitter, the relationship between the dopaminergic signaling network and essential physiological and pathological processes in the nervous systems has become clear. The dopaminergic system is a complex system, organized in parallel and segregated functional streams consisting of motor, reward (limbic), and associative (cognitive) control pathways [186]. However, evidence also exists that the system also exploits integrative mechanisms by which information is transferred between these functional circuits (see [3]). Furthermore, it extensively interacts with other critical signaling pathways [6]. Such a complex intercellular communication occurs through both synaptic and volume transmission (see [64]) and is mediated by a set of GPCRs.

In this respect, extensive evidence has been provided showing that DA receptors can also establish direct allosteric RRIs with other receptor proteins, leading to the formation of receptor complexes and allowing a modulation of signal decoding already at the membrane level and characterized by specific pharmacological profiles; these are potentially of interest to devise new strategies to address relevant disorders. As briefly discussed here, in recent decades, an increasing number of receptor complexes involving DA receptors have been identified and studied. Several aspects, however, remain to be addressed to better understand their function and the possibilities that their targeting may offer.

As previously suggested [157], a first point (of a neuroanatomical nature) we would like to emphasize concerns the need for a more detailed mapping of the different DA-receptor-containing receptor complexes to better understand their distribution in the dopaminergic pathways and to better characterize their location at the cellular level. In this regard, of particular interest would be the study of the descending dopamine pathways, since almost no data concerning the distribution of receptor complexes containing DA receptors in these districts have been obtained so far. A second point (of a pharmacological nature) involves a more detailed assessment of how typical and atypical neuropsychiatric drugs may act on the different receptor complexes in order to optimize existing pharmacological treatments or to develop completely new pharmacological strategies. In this respect, however, the development of receptor-complex-specific ligands appears another very promising strategy. Indeed, the possibility to develop bivalent ligands [187] or to exploit allosteric modulators that are selective for structural domains in the heteroreceptor complexes [129,130] has been demonstrated.

Finally, it should be noted that the research effort to identify and characterize RRIs and receptor complexes has been mainly focused on neurons, given that available data on RRIs and on receptor complexes in astrocytes are more limited. However, a more intense effort in pharmacological research applied to receptor complexes in astrocytes may represent a topic of particular interest, not only to reach a better understanding of the role of neuron–astrocyte crosstalk in dopaminergic systems, but also from a therapeutical standpoint. Such a research effort, indeed, may open the possibility of exploring novel, glia-mediated strategies to address neurodegenerative and functional DA-related disorders (see [141]).

## Figures and Tables

**Table 1:** Dopaminergic pathways [3,6].

Pathway	Description	Functional Features
Nigro-striatal	From the substantia nigra pars compacta to the dorsal striatum	Motor control
Mesolimbic	From the ventral tegmental area to the ventral striatum	Reward-/aversion-related cognition
Mesocortical	From the ventral tegmental area to the prefrontal cortex	Executive functions
Tubero-infundibular	From the hypothalamus to the pituitary gland	Regulation of prolactin secretion
Incerto-hypothalamic	From the zona incerta to the hypothalamus	Visceral and sensorimotor activities
Hypothalamo-spinal	From the hypothalamus to the spinal cord	Modulation of locomotor networks

**Table 2:** Receptor complexes involving dopamine receptors.

Receptor Complex	Cell Location	Reference
A_2A_-D_2_	Neurons Astrocytes	[68] [96]
A_2A_-D_3_	Neurons	[97]
A_2A_-D_4_	Neurons	[98]
NMDA-D_2_	Neurons	[99]
NTS_1_-D_2_	Neurons	[100,101]
CB_1_-D_2_	Neurons	[102]
D_2_-5HT_1_	Astrocytes	[103]
D_2_-5HT_2A_	Neurons	[101,104]
D_2_-OTR	Neurons Astrocytes	[105] [106]
D_2_-GHS_1A_	Neurons	[107]
D_2_-D_4_	Neurons	[108]
α_2A_-D_4_	Neurons	[108]
β_2_-D_4_	Neurons	[109]
D_4_-MOR	Neurons	[110]
CCK_2_-D_2_ (putative)	Neurons	[66,111]
α_1_-D_2_ (putative)	Astrocytes	[112]
A_2A_-D_2_-sigma1	Neurons	[113]
A_2A_-D_2_-mGluR_5_	Neurons	[84]
A_2A_-D_2_-CB_1_	Neurons	[82]
D_1_-D_2_	Neurons	[114]
D_1_-D_3_	Neurons	[115]
A_1_-D_1_	Neurons	[116,117]
NMDA-D_1_	Neurons	[118]
GABA_A_-D_5_	Neurons	[119]
α_1_-D_1_ (putative)	Astrocytes	[112]

NMDA—N-methyl-D-aspartate glutamate receptor; 5HT_1_, 5HT_2A_—type 1 and type 2A serotonin receptors; GHS_1A_—type 1a ghrelin receptor; OTR—oxytocine receptor; CB_1_—type 1 cannabinoid receptor; NTS_1_—type 1 neurotensin receptor; α_1_, β_2_—type α1 and type β_2_ adrenergic receptor; sigma1—sigma1 receptor; mGluR_5_—type 5 metabotropic glutamate receptor; MOR—μ-opioid receptor; type A_1_ and type A_2A_—adenosine receptor.

**Table 3:** Complexes involving dopamine receptors in the ascending dopamine pathways and in neuron–astrocyte crosstalk [108,133,140,141,142].

DA Pathway	Receptor Complexes	Type of Interaction	Location	Major Pathologies
Nigro-striatal	A_2A_-D_2_	Antagonistic	Dorsal striatum	PD
	CB_1_-D_2_	Antagonistic		
	NMDA-D_2_	Antagonistic		
	A_2A_-D_2_-CB_1_	Antagonistic		
	A_2A_-D_2_-mGluR_5_	Antagonistic		
	A_1_-D_1_	Antagonistic		
	D_1_-D_3_	Synergistic		
Mesolimbic	A_2A_-D_2_	Antagonistic	Ventral striatum	Addiction Schizophrenia
	A_2A_-D_3_	Antagonistic		
	NMDA-D_2_	Antagonistic		
	NMDA-D_1_	Antagonistic		
	NTS_1_-D_2_	Antagonistic		
	D_2_-5HT_2A_	Synergistic		
	D_2_-OTR	Synergistic		
	D_1_-D_2_	Signaling cascade change		
	D_1_-D_3_	Synergistic		
	A_2A_-D_2_-sigma_1_	Antagonistic		
	A_2A_-D_2_-mGluR_5_	Antagonistic		
	GABA_A_-D_5_	Antagonistic		
	D_4_-MOR	Synergistic		
Mesocortical	α_2A_-D_4_	Dependent on D_4_ polymorphism	Prefrontal cortex	ADHD
	β_2_-D_4_	Signaling cascade change		
	D_2_-D_4_	Dependent on D_4_ polymorphism		
	A_2A_-D_4_	Antagonistic		
Neuron–Astrocyte crosstalk	A_2A_-D_2_	Antagonistic	Astrocytes	PD Addiction Schizophrenia
	D_2_-OTR	Synergistic		
	D_2_-5HT_1_	Not detailed		

PD, Parkinson’s disease; ADHD, attention-deficit hyperactivity disorder.

## Data Availability

Not applicable.
